# Preoperative systemic immune-inflammation index may predict prolonged mechanical ventilation in patients with spontaneous basal ganglia intracerebral hemorrhage undergoing surgical operation

**DOI:** 10.3389/fneur.2023.1190544

**Published:** 2023-06-15

**Authors:** Huaming Xiao, Lei Li, Feng Zhang, Lei Cheng, Yang Li, Wenlan Han, Huanting Li, Mingchao Fan

**Affiliations:** ^1^Department of Neurosurgery, Weihai Central Hospital, The Affiliated Hospital of Qingdao University, Weihai, Shandong, China; ^2^Department of Neurosurgery, The Affiliated Hospital of Qingdao University, Qingdao, Shandong, China; ^3^Department of Neurosurgical Intensive Care Unit, The Affiliated Hospital of Qingdao University, Qingdao, Shandong, China

**Keywords:** systemic immune-inflammation index, intracerebral hemorrhage, prolonged mechanical ventilation, lactic acid, surgical operation

## Abstract

**Background:**

Prolonged mechanical ventilation (PMV) has been proven as a risk factor for poor prognosis in patients with neurocritical illness. Spontaneous basal ganglia intracerebral hemorrhage (ICH) is one common subtype of hemorrhagic stroke and is associated with high morbidity and mortality. The systemic immune-inflammation index (SII) is used as a novel and valuable prognostic marker for various neoplastic diseases and other critical illnesses.

**Objective:**

This study aimed to analyze the predictive value of preoperative SII for PMV in patients with spontaneous basal ganglia ICH who underwent surgical operations.

**Methods:**

This retrospective study was conducted in patients with spontaneous basal ganglia ICH who underwent surgical operations between October 2014 and June 2021. SII was calculated using the following formula: SII = platelet count × neutrophil count/lymphocyte count. Multivariate logistic regression analysis and receiver operating characteristics curve (ROC) were used to evaluate the potential risk factors of PMV after spontaneous basal ganglia ICH.

**Results:**

A total of 271 patients were enrolled. Of these, 112 patients (47.6%) presented with PMV. Multivariate logistic regression analysis showed that preoperative GCS (OR, 0.780; 95% CI, 0.688–0.883; *P* < 0.001), hematoma size (OR, 1.031; 95% CI, 1.016–1.047; *P* < 0.001), lactic acid (OR, 1.431; 95% CI, 1.015–2.017; *P* = 0.041) and SII (OR, 1.283; 95% CI, 1.049–1.568; *P* = 0.015) were significant risk factors for PMV. The area under the ROC curve (AUC) of SII was 0.662 (95% CI, 0.595–0.729, *P* < 0.001), with a cutoff value was 2,454.51.

**Conclusion:**

Preoperative SII may predict PMV in patients with spontaneous basal ganglia ICH undergoing a surgical operation.

## Introduction

Spontaneous intracerebral hemorrhage (ICH) is one common subtype of hemorrhagic stroke and is associated with high morbidity and mortality (1, 2). The basal ganglia is the usual incidence area of spontaneous ICH, and the most common pathogenic factor is hypertension (3, 4). Postoperative respiratory failure is one of the complications following ICH and is the main cause of mechanical ventilation (5). Among patients with acute central nervous system injury, about 17–33% required endotracheal intubation and mechanical ventilation, and more than 10% of patients with stroke need mechanical ventilation (6), this percentage may be even higher in patients who underwent surgical intervention.

Prolonged mechanical ventilation (PMV) is significantly associated with poor prognosis in patients with critical illness (7, 8). Several risk factors, including old age, obesity, chronic obstructive pulmonary disease, atrial fibrillation, and severe comorbidity, have been identified to be associated with PMV in these patients (9, 10). For patients with hemorrhagic stroke, the risk factors additional include large hematoma volume, obstructive hydrocephalus, coma, brain hernia, and thalamic location of the bleeding (11, 12). PMV is associated with longer hospital stays, increased treatment costs, various mechanical ventilation-related complications, and a relatively high mortality rate (13, 14). Early prediction of PMV may provide useful information for making a comprehensive management plan, help the medical staff to anticipate the ICU course, and help the patient's families to adjust their emotions and live.

Systemic Immune-inflammation index (SII), calculated using lymphocyte, neutrophil, and platelet counts, is a novel comprehensive inflammatory index, and better reflects the state of immune and inflammation of the body. It was reported as a prognostic marker for hepatocellular carcinoma by Hu et al. in 2014 for the first time (15). Recently, many studies have reported that increased SII was associated with unfavorable prognoses in various types of cancers and other critical illnesses (16–18). Best to our knowledge, the SII has not been reported as a predictive factor for PMV, especially for patients with neurocritical illness. We hypothesized that SII can predict PMV in patients with spontaneous basal ganglia ICH who underwent surgical intervention. Thus, the present retrospective study was designed to analyze the predicted value of preoperative SII for PMV in patients with spontaneous basal ganglia ICH who underwent surgical operations.

## Methods

The research protocol was approved by the Human Ethics Committee of the Affiliated Hospital of Qingdao University (QYFY-WZLL-26903) and was registered at the Chinese Clinical Trial Registry (ChiCTR2200056494). The study adhered to the tenets of the Declaration of Helsinki and the related laws and regulations. Informed consent for clinical record data to be used in the study was obtained from the patient's guardians. The privacy rights of human subjects always be observed.

### Patient population

This retrospective cohort study of patients admitted to the Affiliated Hospital of Qingdao University from October 2014 to June 2021 with the diagnosis of spontaneous basal ganglia ICH who underwent surgical intervention. Spontaneous basal ganglia ICH was diagnosed by three senior neurosurgeons based on neuroimaging, history of present illness, and surgical operations results (3, 4).

Patients were included if they met the following criteria: (a) age ≥ 18 years old; (b) unilateral cerebral hemorrhage in the basal ganglia; (c) underwent surgical intervention; (d) the time from onset to surgical operations ≤ 72 h. The exclusion criteria are as follows: (a) with stroke, traumatic brain injury, or craniotomy history; (b) with a history of chronic inflammation status, hematologic (except iron deficiency anemia), or autoimmune diseases; (c) with malignant tumor; (d) need prehospital mechanical ventilation; (e) severe respiratory system diseases, such as chronic obstructive pulmonary disease; (f) non-invasive ventilation; (g) secondary ICH; (h) without complete data.

### Data collection

Clinical data were acquired from the scientific research big data platform and the hospital information system. Surgical intervention was conducted within 72 h of the initial symptoms. All the patients received consistent necessary medical management. The laboratory and clinical data on admission were analyzed and calculated, including age, gender, body mass index, medical history, blood routine analysis, blood biochemical analysis, and Glasgow Coma Scale (GCS). Arterial blood was used for lactic acid analysis. The indications and procedures of operations were under the corresponding surgical guidelines and operating procedures (3, 19). The size of the hematoma was calculated with the following formula: “0.5 × a × b × c,” where “a” and “b” are the largest diameters measured on the CT scans and “c” is the slice thickness (cm) (20).

All patients received general anesthesia and mechanical ventilation during surgical operations. Adjust mechanical ventilation modes and parameters according to actual requirements. The spontaneous breathing trial and weaning were performed based on adequate ventilation indices and oxygenation at the discretion of the neurocritical physician (11). Analgesics-sedatives were achieved with continuous intravenous pumping at the discretion of the treating physician. Depth of sedation was controlled at −1 – −2 score (Richmond Agitation Sedation Scale).

The SII was calculated using the following formula: SII = platelet count × neutrophil count/lymphocyte count (15). PMV was defined as mechanical ventilation lasting for more than 24 h after surgical operations (21, 22). Length of mechanical ventilation was defined as the time from initiation of ventilatory to the accomplishment of weaning. The prognosis of patients at 3 months was evaluated using the modified Rankin Scale (mRS). The score of 0–2 was a favorable prognosis, while 3–6 was defined as an unfavorable prognosis.

### Statistical analysis

Statistical analysis was performed using IBM SPSS Statistics 24.0 (SPSS Inc., Chicago, Illinois, USA). Continuous variables were summarized as means ± standard deviations (SD). The abnormal distribution of continuous variables was summarized as median and interquartile ranges (25^th^ to 75^th^ percentile). Categorical data were presented as frequency and percentage. The student's *t*-test and Kruskal-Wallis test were used to compare the normal distribution and abnormal distribution continuous variables respectively. The Chi-square test was used to compare the categorical data. Logistic regression analysis was conducted to identify the independent risk factors. Receiver operating characteristic curve (ROC) analysis was performed to define the differences in the area under the curves (AUC) and the cutoff value, then the sensitivity and specificity. A *P* value < 0.05 was considered statistically significant.

Due to SII being calculated by multiplying the neutrophil and platelet count and then dividing the result by the lymphocyte count, leukocyte count and neutrophil count were abandoned in the logistic regression models in order to avoid bias effect.

## Results

### Baseline characteristics

**Figure 1:**
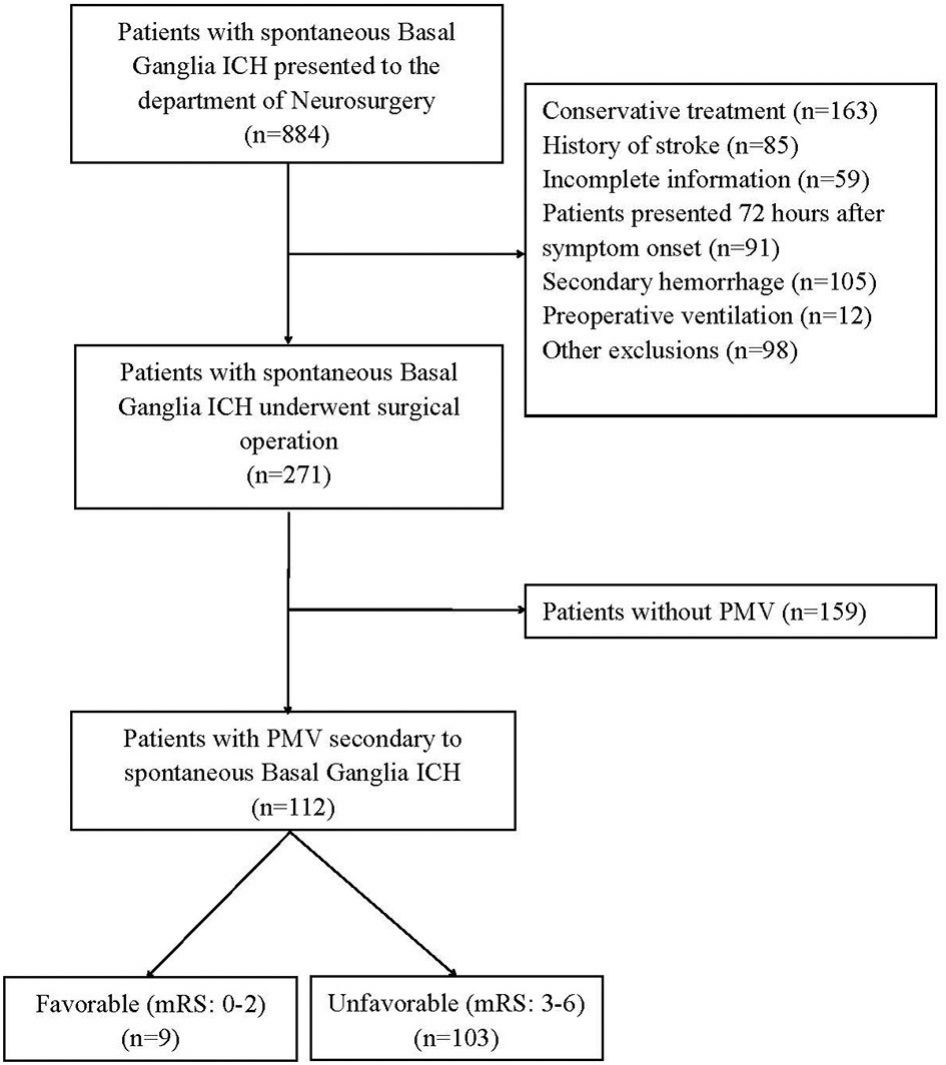
The study flow chart. ICH, Intracerebral Hemorrhage; PMV, Prolonged Mechanical Ventilation; mRS, the Modified Rankin Scale.

The demographics and details information of all patients was shown in Table 1. A total of 271 patients were enrolled. The median age of these patients was 56 years (interquartile range, 47–65 years), and 80 patients (29.52%) were women. Of these, 112 patients (47.6%) who presented PMV were allocated to the PMV group, and 159 (52.4%) patients without PMV were allocated to the non-PMV group (Figure 1). Percutaneous tracheotomy was performed in 67 (59.82%) of 112 patients with PMV. The median time of tracheotomy was 6 (5–9) days after surgical operation. Of these 271 patients, 106 patients had a favorable prognosis (39.1%), and 165 patients (60.9%) had an unfavorable prognosis.

**Table 1:** The demographic and baseline characteristics of patients in the PMV and non-PMV groups.

**Variable**	**Total (*n* = 271)**	**PMV**		***P*-value**
		**YES (*n* = 112)**	**NO (*n* = 159)**	
Gender (female, %)	80 (29.52)	32 (28.57)	48 (30.19)	0.714
Age (years)	56 (47–65)	56 (47–67)	57 (47–65)	0.513
BMI	25.40 (23.30–27.68)	25.40 (23.40–27.70)	25.40 (22.90–27.55)	0.656
Smoking (%)	71 (26.20)	32 (28.57)	39 (24.53)	0.502
Drinking (%)	76 (28.04)	31 (27.68)	45 (28.30)	0.850
Cardiac insufficiency (%)	30 (11.07)	17 (15.18)	13 (8.18)	0.070
Hypertension (%)	176 (64.94)	78 (69.64)	98 (61.64)	0.234
Diabetes mellitus (%)	23 (8.49)	12 (10.71)	11 (6.92)	0.287
Systolic pressure (mmHg)	158 (140–180)	163 (142–190)	155 (140–179)	**0.043**
Diastolic pressure (mmHg)	92 (80–105)	94 (80–110)	92 (80–101)	0.782
Length of ICU stay (days)	3 (1–9)	9 (3–19)	1 (0–3)	**<0.001**
Length of stay (days)	16 (11–25)	23 (14–36)	14 (11–18)	**<0.001**
GCS	9 (7–12)	8 (5–9)	10 (8–13)	**<0.001**
Hematoma size (ml)	50 (40–80)	70 (50–80)	50 (40–60)	**<0.001**
Intraventricular hematoma	121 (44.65)	55 (49.11)	57 (35.85)	0.215
Surgical Operation				0.056
Craniectomy (%)	121 (44.65%)	59 (52.68%)	62 (38.99%)	
Endoscopic (%)	86 (31.73%)	33 (29.46%)	53 (33.33%)	
Burr hole aspiration (%)	64 (23.62%)	20 (17.86%)	44 (27.67%)	
Serum potassium (mmol/L)	3.77 ± 0.52	3.64 ± 0.55	3.86 ± 0.49	**<0.001**
serum sodium (mmol/L)	140 (137–142)	140 (137–142)	140 (137–142)	0.477
Serum albumin (g/L)	39.20 ± 5.30	38.26 ± 5.62	39.86 ± 4.98	**0.016**
Monocyte (× 10^9^/L)	0.54 (0.38–0.75)	0.57 (0.39–0.82)	0.54 (0.38–0.73)	0.583
Neutrophil (× 10^9^/L)	9.86 (7.10–13.01)	11.53 (8.37–14.55)	8.61 (6.43–11.37)	**<0.001**
Lymphocyte (× 10^9^/L)	1.11 (0.78–1.66)	1.07 (0.71–1.56)	1.21 (0.85–1.76)	0.077
WBC (× 10^9^/L)	11.93 (9.31–14.95)	13.00 (10.47–16.39)	10.56 (8.18–13.35)	**<0.001**
Hemoglobin (g/L)	146 (132–157)	147 (131–159)	146 (134–157)	0.906
Platelet (× 10^9^/L)	211 (178–251)	214 (184–255)	207 (170–248)	0.130
SII	1,796.35 (1,093.86–3,047.16)	2,463.14 (1,369.03–3,516.28)	1,538.17 (980.51–2,432.10)	**<0.001**
Blood glucose (mmol/L)	7.8 (6.8–9.3)	8.3 (7.3–10.2)	7.4 (6.3–8.6)	**<0.001**
Lactic acid (mmol/L)	1.5 (1.0–2.0)	1.7 (1.1–2.4)	1.2 (1.0–1.5)	**<0.001**
Uric acid (μmol/L)	226.5 (170.1–285.0)	225 (174–301)	228 (168–264)	0.234
Lactate dehydrogenase (u/L)	196.5 (164.0–249.0)	205 (168–258)	194 (158–243)	**0.046**
Postoperative analgesics-sedatives	142 (52.40)	66 (58.93)	76 (47.80)	0.071

BMI, body mass index; GCS, Glasgow coma scale; SII, systemic immune inflammation index; WBC, white blood cells. The significance of bold data: *P* < 0.05.

### The association of PMV with prognosis

Among these 112 patients with PMV, 9 cases (8.04%) had good prognosis and 103 patients (91.96%) had poor (χ^2^ = 77.422, *P* < 0.001). The duration of PMV was 120 (48–258) h. A total of 22 patients (8.12%) died during hospitalization and follow-up, 18 patients (16.07%) were in the PMV group, and 4 patients (2.52%) were in the non-PMV group (χ^2^ = 16.189, *P* < 0.001). The length of ICU stay (*P* < 0.001) and total hospital stay (*P* < 0.001) in PMV group patients were significantly longer than those in the non-PMV group. PMV was a risk factor for poor prognosis of patients with spontaneous basal ganglia ICH who underwent surgical operations [Odds Ratio (OR), 17.905; 95% confidence interval (CI), 8.440–37.985; *P* < 0.001].

### Univariate analysis of pre-operations risk factors for PMV

The results of the univariate analysis of risk factors for PMV are presented in Table 1. Univariate analysis revealed that systolic pressure (*P* = 0.043), GCS (*P* < 0.001), hematoma volume (*P* < 0.001), serum Potassium (*P* < 0.001), blood glucose (*P* < 0.001), serum albumin (*P* = 0.016), and lactic acid (*P* < 0.001) on admission were significantly correlated with PMV. There was no relationship between age (*P* = 0.550), gender (*P* = 0.714), Body Mass Index (*P* = 0.656), history of smoking (*P* = 0.502), history of drinking (*P* = 0.850), history of diabetes (*P* = 0.287), serum sodium (*P* = 0.477), intraventricular hematoma (*P* = 0.215), Analgesics-sedatives (*P* = 0.071), and PMV.

Of these 271 patients, 121 patients (44.65%) underwent craniectomy, 86 patients (31.73%) underwent endoscopic evacuation of hematoma, and 64 patients (23.62%) underwent directional burr hole hematoma aspiration. There was no significant difference in postoperative prolonged mechanical ventilation among different surgical operations (*P* = 0.056).

Of the peripheral blood cells analysis, neutrophil (*P* < 0.001) and WBC (*P* < 0.001) were significantly correlated with PMV. And monocyte (*P* = 0.287), lymphocyte (*P* = 0.077), hemoglobin (*P* = 0.906), and platelet (*P* = 0.130) didn't find a significant correlation with PMV. The SII of PMV group patients was significantly higher than that of non-PMV patients (*P* < 0.001).

### Logistic regression analysis of predictive factors for PMV

**Figure 2:**
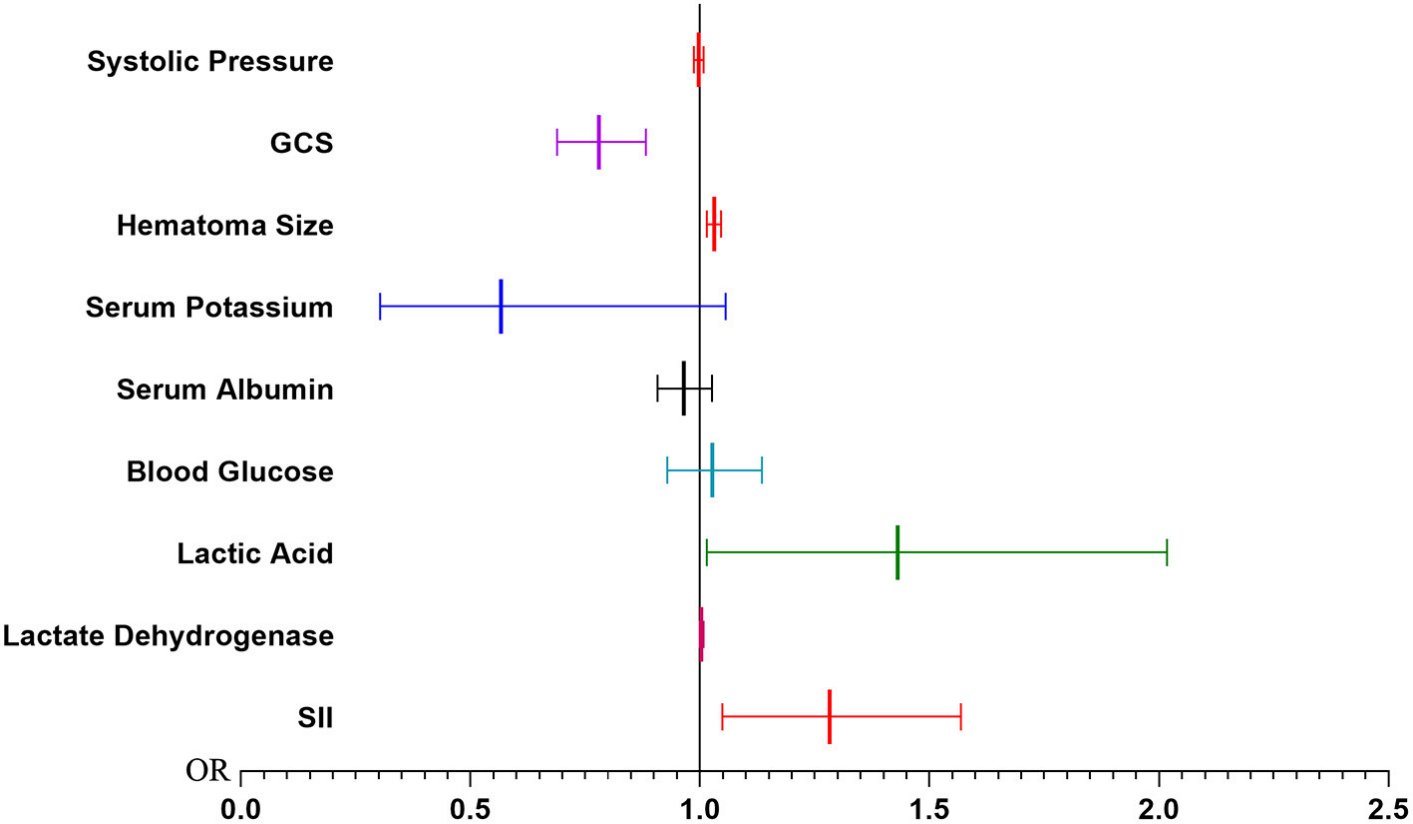
Forest map of multivariate logistic regression. SII, Systemic Immune Inflammation Index.

The results of univariate and multivariate Logistic regression analysis were presented in Table 2. These risk variables, except the neutrophil and WBC, were analyzed using univariate Logistic regression, and all of them were statistically significant. Then these factors with statistical significance in univariate logistic regression were brought into a multivariate logistic regression analysis to establish the PMV risk prediction model. We found that GCS (OR, 0.780; 95% CI, 0.688–0.883; *P* < 0.001), hematoma size (OR, 1.031; 95% CI, 1.016–1.047; *P* < 0.001), lactic acid (OR, 1.431; 95% CI, 1.015–2.017; *P* = 0.041), and SII (OR, 1.283; 95% CI, 1.049–1.568; *P* = 0.015) were significant in multivariate logistic regression (Figure 2).

**Table 2:** Univariate and multivariate regression analysis of factors related to PMV.

**Predictors**	**Univariate analysis**		**Multivariate analysis**	
	**OR (95% CI)**	***P*** **-value**	**OR (95% CI)**	***P*** **-value**
Systolic pressure	1.008 (1.000–1.016)	0.043	0.998 (0.987–1.008)	0.679
GCS	0.685 (0.616–0.762)	<0.001	**0.780 (0.688–0.883)**	**<0.001**
Hematoma size	1.041 (1.028–1.055)	<0.001	**1.031 (1.016–1.047)**	**<0.001**
Serum potassium	0.415 (0.251–0.687)	0.001	0.566 (0.303–1.056)	0.074
Serum albumin	0.942 (0.898–0.988)	0.013	0.965 (0.908–1.026)	0.249
Blood glucose	1.150 (1.051–1.258)	0.002	1.028 (0.930–1.136)	0.594
Lactic acid	1.848 (1.375–2.482)	<0.001	**1.431 (1.015–2.017)**	**0.041**
Lactate dehydrogenase	1.005 (1.001–1.008)	0.006	1.004 (1.000–1.008)	0.067
SII	1.471 (1.235–1.752)	<0.001	**1.283 (1.049–1.568)**	**0.015**

GCS, Glasgow coma scale; SII, systemic immune inflammation index. The significance of bold data: *P* < 0.05.

### ROC curves analysis of predictive factors for PMV

**Figure 3:**
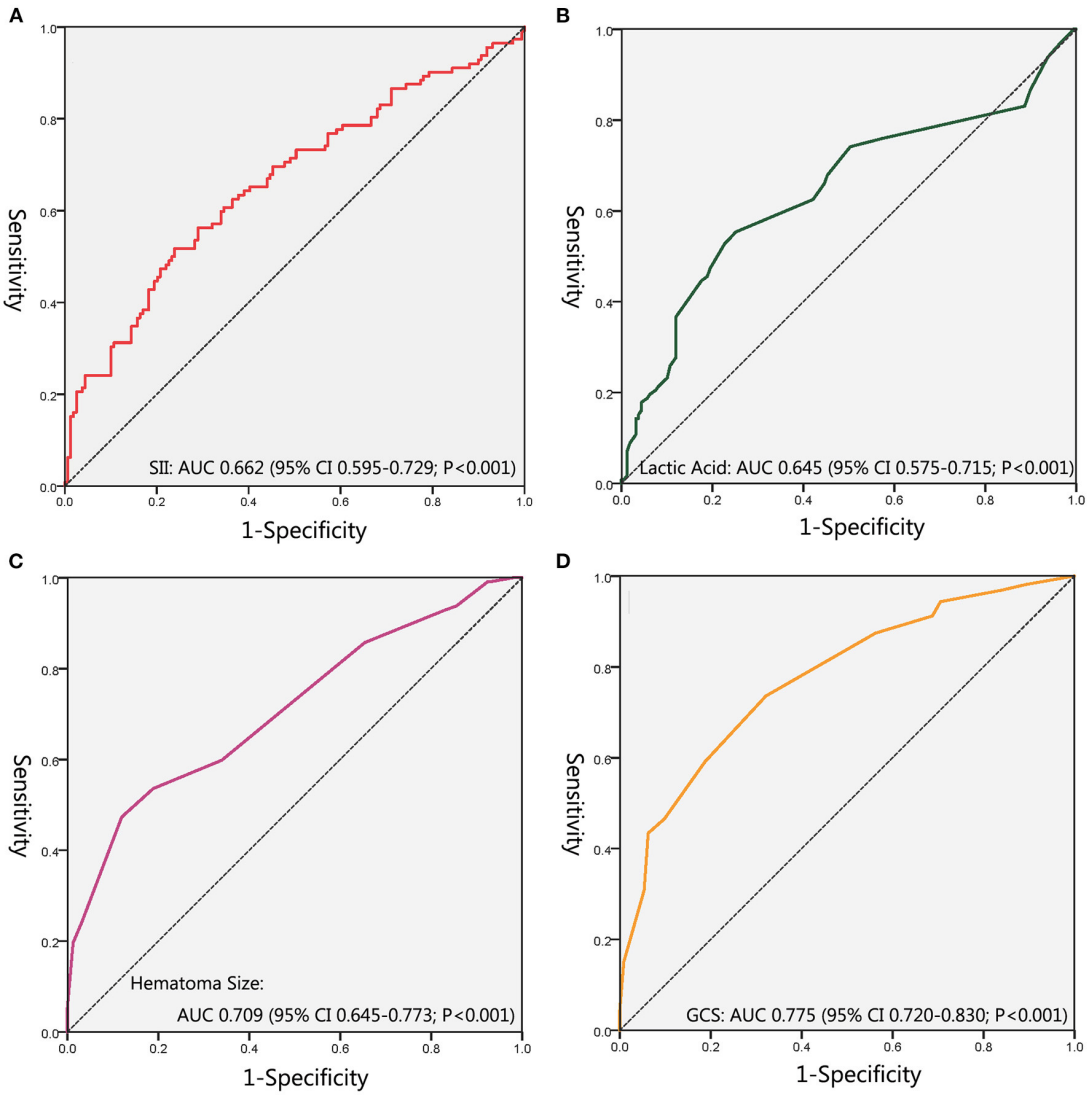
The receiver-operating characteristic curves. **(A)** ROC for SII; **(B)** ROC for Lactic Acid; **(C)** ROC for Hematoma Size; **(D)** ROC for GCS. SII, Systemic Immune Inflammation Index; ROC, Receiver Operating Characteristics Curve; GCS, Glasgow Coma Scale.

ROC curves were performed to evaluate the predictive ability of SII, lactic acid, hematoma size, and GCS (Figure 3; Table 3). The corresponding AUC of SII was 0.662 (95% CI, 0.595–0.729, *P* < 0.001), and the cutoff value was 2,454.51, with sensitivity and specificity of 51.8% and 76.1%, respectively. The corresponding AUC of lactic acid was 0.645 (95% CI, 0.575–0.715, *P* < 0.001), and the cutoff value was 1.55 with sensitivity and specificity of 55.4% and 74.8%, respectively. The corresponding AUC of hematoma size was 0.709 (95% CI, 0.645–0.773, *P* < 0.001), and the cutoff value was 75 ml with sensitivity and specificity of 47.3% and 88.1%, respectively. The corresponding AUC of GCS was 0.775 (95% CI, 0.720–0.830, *P* < 0.001), and the cutoff value was 8.5 with sensitivity and specificity of 73.6% and 61.9%, respectively.

**Table 3:** Diagnostic values of factors related to PMV.

**Variable**	**AUC (95% CI)**	***P*-value**	**Cutoff value**	**Sensitivity**	**Specificity**
SII	0.662 (0.595–0.729)	<0.001	2,454.51	0.518	0.761
GCS	0.775 (0.720–0.830)	<0.001	8.5	0.736	0.619
Lactic acid	0.645 (0.575–0.715)	<0.001	1.55	0.554	0.748
Hematoma size	0.709 (0.645–0.773)	<0.001	75	0.473	0.881

Based on the SII cutoff value, these 271 patients were then dichotomized into 2 groups, SII <2,454.51 group (*n* = 182, 67.16%) and SII > 2,454.51 group (*n* = 89, 32.84%). The prognosis was poorer for SII > 2,454.51 group patients than for SII <2,454.51 group patients (χ^2^ = 50.502, *P* < 0.001). However, there was no statistically significant difference in mortality between these two groups (χ^2^ = 3.196, *P* = 0.074).

## Discussion

In this retrospective study, We retrieved and analyzed the clinical data of patients with spontaneous basal ganglia ICH who underwent surgical operations in a single center during an 8-year period using the hospital's scientific research big data platform. For the first time, we examined the risk factors of postoperative PMV after spontaneous basal ganglia ICH and analyzed the association between SII and PMV. We found that preoperative SII may predict PMV in patients with spontaneous basal ganglia ICH who underwent surgical operations. These findings may be helpful for the medical staff to anticipate the therapy course, and help the patient families to adjust their emotions and face the challenge of life.

The time definition of PMV is still uncertain, ranging from 24 h to 21 days (11, 12, 14, 21–23). This is mainly based on the evaluation criteria of each clinical center, and the actual needs of the prognosis estimate (24). The length of PMV defined by the surgical department was shorter, while the PMV time defined by the rehabilitation medicine department tended to be longer. PMV was defined as mechanical ventilation for more than 24 h in this study, and the clinical prognostic differentiation was satisfactory. PMV has been proven to harm the prognosis. A high incidence of PMV (47.6%) was shown in our cohort, and more than 90% of them had unfavorable prognoses. The length of ICU stay and total length of stay in PMV patients were significantly longer, and the mortality was higher.

Acute persistent disturbance of consciousness and the related inability to protect the airway, and abnormal respiratory mechanics were secondary to severe intracranial lesions are the most frequent reasons for initiating mechanical ventilation and postoperative weaning difficulty (6, 25–27). Among the factors associated with unfavorable prognosis of neurocritical ill patients, GCS and hematoma size are general acceptance predictors (12, 13). Decreased consciousness was often significantly associated with increased intracranial hematoma volume (28). The surgical operation usually signifies large intracranial bleeding and severe disease progression. In the present study, initial GCS <9 and hematoma size > 75 ml were identified as significant and independent predictors of PMV in patients with spontaneous basal ganglia ICH who underwent surgical operations.

The inflammation is implicated in the pathogenesis of stroke, and the inflammatory response following acute brain injury has been demonstrated (27, 29). The extent of the systemic inflammatory response and count of circulating neutrophils were significant correlation with hematoma size (30). Increased circulating neutrophil is recruited directly due to the inflammatory environment and high level of the stress state. Catecholamine released from sympathetic activation also leads to neutrophil increase and lymphopenia (31). Lymphopenia on admission is one of the characteristics of neurogenic immunosuppression, independently associated with the severity and extension of brain injury, and reflects the functional frailty status of the patient (32, 33). In our study, the lymphocyte count of patients in the PMV group was lower than those in the non-PMV group (1.07 × 10^9^/L vs. 1.21 × 10^9^/L), but the difference was not statistically significant (*P* = 0.077). In addition to preventing bleeding and promoting hemostasis, the platelet participates in post-stroke immune-inflammatory responses by releasing chemokines and cytokines (34, 35). In the present study, we found that the platelet count of patients in the PMV group was higher than those in the non-PMV group (214 × 10^9^/L vs. 207 × 10^9^/L), but the statistical results were not significant too (*P* = 0.130).

The systemic production of inflammatory mediators induces inflammatory responses not only in the brain environment but also in a systemic inflammatory environment. Excessive systemic inflammatory response participates in the pathogenesis of acute lung injury (36, 37). Sympathetic overstimulation and large amounts of catecholamines release into the systemic circulation increase pulmonary vascular hydrostatic pressure and endothelial permeability, causing neurogenic pulmonary edema (38, 39). SII, which was obtained by combining neutrophils, lymphocytes, and platelets in a single index, has been confirmed as a novel predictive inflammation marker of PMV in our cohort, and the cut-off value was 2,454.51. To our knowledge, the present report may be the first study to show that pre-operation increased SII can be a risk factor for PMV in patients with spontaneous basal ganglia ICH who underwent surgical operations.

In this study, another risk factor identified for PMV was lactic acid. Elevated lactic acid underscores the systemic impact of intracranial pathology, and predicts the disease's systemic severity in patients with devastating neurologic diseases (40). Due to excessive catecholamine release could induce increased glucose metabolism with a rapid output of lactate in the systemic circulation, and enhanced renal perfusion may lead to negative liquid equilibrium (41, 42). In addition, a sharp increase in intracranial pressure leads to the use of large doses of osmotic diuretics, which may cause subsequential hypovolemia. Other potential causes of elevated lactic acid include neurogenic pulmonary edema and acute lung injury (43). But lactic acidosis is multifactorial, a prospective study with other causes excluded may be useful.

In the present study, We found that pre-operation SII predicted PMV quite well in patients with spontaneous basal ganglia ICH who underwent surgical operations. The incorporation of these cheap and readily available markers into clinical treatment may simplify prediction procedures and strengthen the predictive power. However, this article also has some limitations. First, it is a single-center retrospective study with a relatively small scale and may have potential biases. Second, although we excluded comorbidities that might affect inflammation, immunity, and breathing, some potential confounders may not be easily detected. Third, the purpose of this study was to analyze the predictive value of preoperative factors for PMV, but some factors after surgery were also affecting PMV (such as acute respiratory distress syndrome or hypostatic pneumonia), and no further comparison was made in this study. Fourth, we only validated the effectiveness of preoperative SII in predicting PMV after surgical operations for spontaneous basal ganglia ICH but did not cross-compare SII with other inflammatory indices. Fifth, only these patients with spontaneous basal ganglia intracerebral hemorrhage who underwent surgical treatment were included and analyzed, so the information on patients who underwent conservative treatment could not be presented. Finally, we only observed the baseline SII on admission, the relationship between the dynamic changes of SII and the occurrence of PMV worthy of further study.

## Conclusion

Preoperative SII may predict PMV in patients with spontaneous basal ganglia ICH who undergo surgical operations.

## Data availability statement

The raw data supporting the conclusions of this article will be made available by the authors, without undue reservation.

## Ethics statement

The studies involving human participants were reviewed and approved by the Human Ethics Committee of the Affiliated Hospital of Qingdao University (QYFY-WZLL-26903). The patients/participants provided their written informed consent to participate in this study.

## Author contributions

HX: conceptualization, data curation, formal analysis, investigation, methodology, visualization, and writing—original draft. MF: conceptualization, data curation, formal analysis, investigation, methodology, visualization, writing—original draft, and writing—review and editing. HL: conceptualization, formal analysis, investigation, methodology, visualization, and writing—review and editing. WH and YL: data curation and writing—review and editing. LC: data curation, formal analysis, and writing—review and editing. FZ: data curation, visualization, and writing—review and editing. LL: conceptualization, data curation, formal analysis, investigation, methodology, project administration, visualization, writing—original draft, and writing—review and editing. All authors contributed to the article and approved the submitted version.
